# Spectroscopy, Morphology, and Electrochemistry of Electrospun Polyamic Acid Nanofibers

**DOI:** 10.3389/fchem.2021.782813

**Published:** 2022-02-16

**Authors:** Siyabulela Hamnca, Jessica Chamier, Sheila Grant, Timothy Glass, Emmanuel Iwuoha, Priscilla Baker

**Affiliations:** ^1^ SensorLab, Chemistry Department, University of the Western Cape, Bellville, South Africa; ^2^ HySA Catalysis, Department of Chemical Engineering, University of Cape Town, Rondebosch, Cape Town, South Africa; ^3^ Chemistry Department, University of Missouri, Columbia, MO, United states

**Keywords:** polyamic acid, nanofibers, screen-printed carbon electrodes, electrochemistry, electrospun (ES) nanofibers

## Abstract

Polyamic acid (PAA) nanofibers produced by using the electrospinning method were fully characterized in terms of morphology and spectroscopy. A PAA nanofiber–modified screen-printed carbon electrode was applied to the detection of selected sulfonamides by following an electroanalytical protocol. The polyamic acid (PAA) nanofibers were characterized using Fourier transform infrared (FTIR) spectroscopy to study the integrity of polyamic acid functional groups as nanofibers by comparing them to chemically synthesized polyamic acid. A scanning electron microscope (SEM) was used to confirm the morphology of the produced nanofibers and 3D arrangement at the electrode interface. The Brunauer–Emmett–Teller (BET) method was used to determine the surface area of the nanofibers. Atomic force microscopy (AFM) was used to study the porosity and surface roughness of the nanofibers. Electrochemical evaluation based on diffusion-controlled kinetics was applied to determine the number of electrons transferred in the system, the surface concentration of the deposited PAA thin film (2.14 × 10^−6^ mol/cm^2^), and the diffusion coefficient (D_e_) for the PAA nanofiber–modified screen-printed carbon electrode (9.43 × 10^−7^ cm^−2^/s). The reported LODs for sulfadiazine and sulfamethazine detection are consistent with requirements for trace-level monitoring by early warning diagnostic systems.

## Introduction

Electrospinning is an efficient and highly scalable method used for preparation and production of a variety of nanostructured polymer materials. The process of electrospinning requires the application of controlled voltage to produce fibers from charged polymer solutions producing fibers with diameters ranging from micron to nanometer size (Frenot and Chronakis, 2003; Valizadeh and Mussa Farkhani, 2013; Sapountzi et al., 2017; Chen et al., 2019). These polymer nanofibers exhibit unique properties such as high surface area-to-volume ratio, good structural and mechanical properties, extreme flexibility, low basic weight, and cost effectiveness (Machotová et al., 2016). Electrospinning works well for polymers with high molecular weight and good solubility to yield concentrated solutions. Additionally, polymers should also have good conductivity and viscosity. However, finding the correct mix of polymer solution properties for efficient spinning remains a challenging exercise. Researchers have adopted different approaches to improve the spinnability of polymers and other non-polymer materials, employing carrier polymers or additives such as surfactants to impose spinning. Aduba et al. (2015) effectively used a carrier polymer method for producing electrospun nanocomposite fibers from PEGylated PAMAM dendrimers, blended with a small amount of high–molecular weight polyethylene oxide (PEO). Saquing et al. (2013) was able to produce high-quality spun fibers of alginate by blending it with high–molecular weight polyethylene oxide (PEO). The molecular weight of the polymer affects the polymer or polymer composite solution viscosity, surface tension, and conductivity (Machotová et al., 2016).

Polyamic acid (PAA) is a polyimide precursor polymer with amide and carboxylic functional groups. PAA is easy to synthesize and process due to its high solubility in aprotic solvents. PAA polymer thin films may easily be produced *in situ* at Au and glassy carbon electrode surfaces and functionalized in various ways to prepare very efficient electrochemical sensors (Andreescu et al., 2005; Noah et al., 2012; Hess et al., 2014; Hamnca et al., 2016). While electrochemical polymerization yields nanostructured PAA, no evidence of organized nanofibers produced from chemically synthesized PAA powder by any method has been reported yet. In this study, the semiconducting polymer, polyamic acid (PAA), was used to produce nanofibers by electrospinning PAA from a homogeneous blended polymer solution, using a minimal amount of high–molecular weight polyvinylpyrrolidone (PVP) for efficient nanofiber production, with good processability and yield.

Electrospinning of continuous fibers is improved by chemical cross-linkers, additives, and carrying polymers when spinning low–molecular weight polymers such as PAA (Wang et al., 2016). Polyvinylpyrrolidone (PVP) has been widely reported as an efficient carrier polymer in spinning of a wide range of materials, due to its high solubility in aprotic solvent such as dimethylformamide (DMF) and N,N-dimethylacetamide (DMAc). Lubasova et al. (2015) used PVP and PVP/poly (acrylic acid) blend to produce hydrogel nanofibers simply by heat treatment of the electrospun nanofibers without the inclusion of any toxic agent for cross-linking as reported by the authors. PVP has also been used as a carrying polymer in the fabrication of titania nanofibers (Chandrasekar et al., 2009). In this study, we present the spectroscopy, morphology, and electrochemistry of polyamic acid (PAA) nanofibers. The effect of carrier polymer loading, applied voltage, and flow rate on the efficacy of nanofiber formation was evaluated. Sulfonamides are a class of broad-spectrum synthetic bacteriostatic antibiotics which are usually administered in animal husbandry to prevent and control diseases. Sulfonamides are classified as the emerging contaminants in the aquatic environment, for which there are no regulatory guidelines. Therefore, it is important to develop tools that detect and report these antibiotics at trace concentrations. As contaminants of emerging concern, sulfonamides are frequently detected in all kinds of environmental water, surface water, ground water, and wastewater systems. Real-time detection of sulfonamides and other pharmaceuticals in the environment remains a global objective. Electrochemical sensors and biosensors play a significant role in delivering data in a simple, portable, and cost-effective format.

## Experimental

### Chemicals and Reagents

The reagents 4, 4-oxydianiline (97%), 1, 2, 4, 5-benzenetetracarboxylic anhydride (97%), acetonitrile (99%), N,N-dimethylacetamide (DMAc), dimethylformamide (DMF), trizma–hydrochloric acid (Tris–HCl), polyvinylpyrrolidone (MW. 130000 g/mol), sulfadiazine (99%), and sulfamethazine (99%) were all obtained from Sigma-Aldrich, South Africa. All chemicals were of analytical grade and used without further purification. Deionized (ultra-pure) water purified at a resistivity of 18.2 MΩ/cm. A Milli-QTM system (Millipore) was used for preparation of all aqueous solutions.

### Polyamic Acid Synthesis and Electrospinning of Polyamic Acid

Polyamic acid was synthesized from organic solvents, using 4, 4-oxydianiline and benzene 1,2,4,5-tetracarboxylic anhydride precursors, as previously described in the literature (Andreescu et al., 2005; Noah et al., 2012; Hamnca et al., 2016). A blend of polyamic acid (12% by wt.) and PVP (3% by wt.) was prepared using dimethylformamide (DMF) and dimethylacetamide (DMAc) as solvents, respectively. The elctrospinning of polyamic acid (PAA) was carried out using a syringe of a diameter of 0.5 mm at an applied voltage of 16–16.8 kV, depending on humidity. The electrospinning experiments were performed at room temperature (typically 23°C) with atmospheric humidity in the range of 21–24%. The flow rate ranged between 99 and 150 μl/h, with the spinneret and collector distance set at 15 cm. The flow rate and spinning time had a direct effect on the amount of electrospun fibers produced. The deposited and freestanding nanofibers produced were dried and stored at room temperature. Freestanding PAA nanofibers were produced using a custom-designed electrospinning instrument in the biological engineering department (University of Missouri, Columbia, MO, United States). The freestanding nanofibers were characterized using microscopic and thermogravimetric techniques.

### Instrumentation

An electrospinning instrument from IME technologies situated in the chemical engineering department (HySA) at the University of Cape Town was used to produce nanofibers. Fourier transform infrared spectroscopy was carried out using a PerkinElmer Spectrum 100 instrument. Thermal stability data for freestanding PAA nanofibers were obtained by using thermogravimetric analysis (TGA) Q500 V3.13 Build 261 in the temperature range of 10–475°C at a heating rate of 10°C/min in atmospheric nitrogen. The morphology studies were conducted on a Hitachi S3000N scanning electron microscope (SEM) and Zeiss Auriga high-resolution field emission gun (fegsem) scanning electron microscope (HRSEM) for samples deposited onto a carbon grid. Electrochemical deposition and evaluation were performed using a PalmSensPTrace 4.4 workstation (Bioanalytical Systems, United States). The electrodeposited thin films and electrospun nanofibers were prepared on screen-printed carbon electrodes from DropSens (DRP-C110, Spain). Nitrogen sorption experiments were performed on a Micromeritics TrisStar II 3020 version 2.0 instrument. The surface area was calculated from the Brunauer–Emmett–Teller (BET) equation. The Nanosurf EasyScan2 was used with highly doped silicon material probing tips with a resistivity of 0.01–0.02 (from NANOSENSORS™). Atomic force microscopy (AFM) was used to study the average height distribution of the screen-printed electrodes modified with polyamic acid nanofibers.

### Electrochemical Evaluation

Scan rate–dependent cyclic voltammetry (CV) was used to evaluate the electrochemical integrity of the deposited PAA materials and, subsequently, its analytical response toward the quantification of a selection of sulfonamides. Scan rates ranged from 10 to 100 mV/s, with a potential window set between −1,000 and +1,000 mV vs. Ag/AgCl in a 0.1 M phosphate buffer (pH 7) electrolyte. The electrochemical determinations of sulfonamides were done in 0.1 M Tris–HCl (pH 8) using square wave voltammetry (SWV) at a scan rate of 50 mV/s, which was achieved by setting the frequency to 10 Hz and the step potential to 5 mV vs. Ag/AgCl. The electrochemical measurements were performed using the three-electrode screen-printed system cell, that is, working electrode (area = 0.13 cm^2^) and auxiliary (carbon) and reference electrodes (Ag/AgCl). The SPCE was inserted into 3 ml electrolyte solution which was bubbled with argon gas to remove dissolved oxygen. The working electrode surface was activated by five scans using the cyclic voltammetry technique at 50 mV/s, prior to data collection in electrochemical characterization of material and evaluation of the selected analyte. These instrumental parameters were consistently applied to all electrochemical experiments.

## Results and Discussion

The solubility of PAA was tested in phosphate buffer, DMF, DMac, acetic acid, chloroform water, dimethyl sulfoxide (DMSO), acetone, NMP, acetonitrile (ACN), tetrahydrofuran (THF), methanol, chloroform, diethyl ether, and tetrahydrofuran/methanol (1:1 ratio) at concentrations 1, 5, 10, and 20% wt. PAA. However, polyamic acid was found to be completely soluble in DMF and DMAc, which are high–boiling point aprotic solvents (153°C and 165.1°C, respectively) (see Supplementary Table S1.1).

**FIGURE 1:**
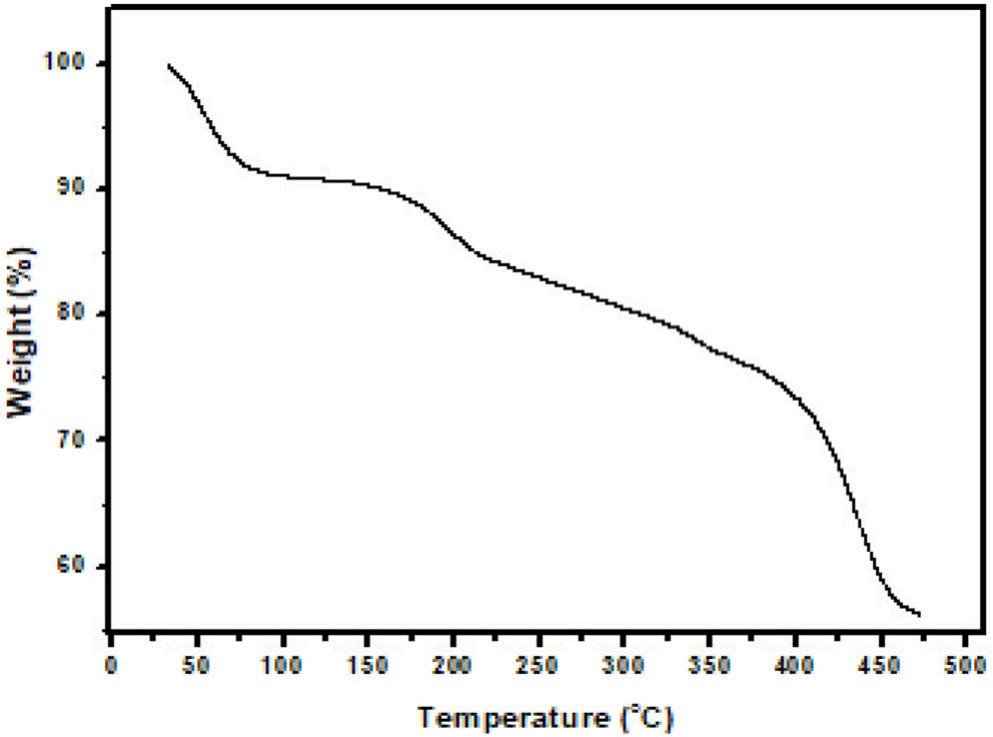
Thermogravimetric analysis (TGA) curve of PAA nanofibers recorded under nitrogen atmosphere up to 475°C at 10°C/min.

Chemically synthesized PAA did not provide solutions in DMF and DMAc for spinning on its own. Low concentrations of PVP (1, 2, 3, and 5% by wt.) were added to 12% by wt. PAA solutions using DMF and DMAc, respectively. Each of these solutions were tested in terms of the quality of nanofibers that could be produced under the optimized instrumental parameters. The optimal spinning solution was produced from 12% by wt. PAA and 3% by wt (see Supplementary Table S1.2). PVP dissolved in DMF yielded fine nanofibers with minimal beading. The morphology of freestanding PAA nanofibers produced from the custom-made electrospinning instrument (University of Missouri, United States) was determined by a scanning electron microscope (SEM), and thermogravimetric analysis (TGA) was used to study the thermal stability of the fibers. The diameters of the bead-free PAA fibers varied from 100 nm to 100 µm (see Supplementary Figure SI.1). The thermal degradation pathway showed a small change in weight percentage (3.57%) at 53°C which was attributed to water and solvent (DMF) evaporation. At a higher temperature (194°C), a further weight loss (12.76%) was observed and was assigned to water loss resulting in imidization of PAA. Above 300°C, no significant weight loss steps were observed (Figure 1).

### Fourier Transform Infrared Spectroscopy Characterization

**FIGURE 2:**
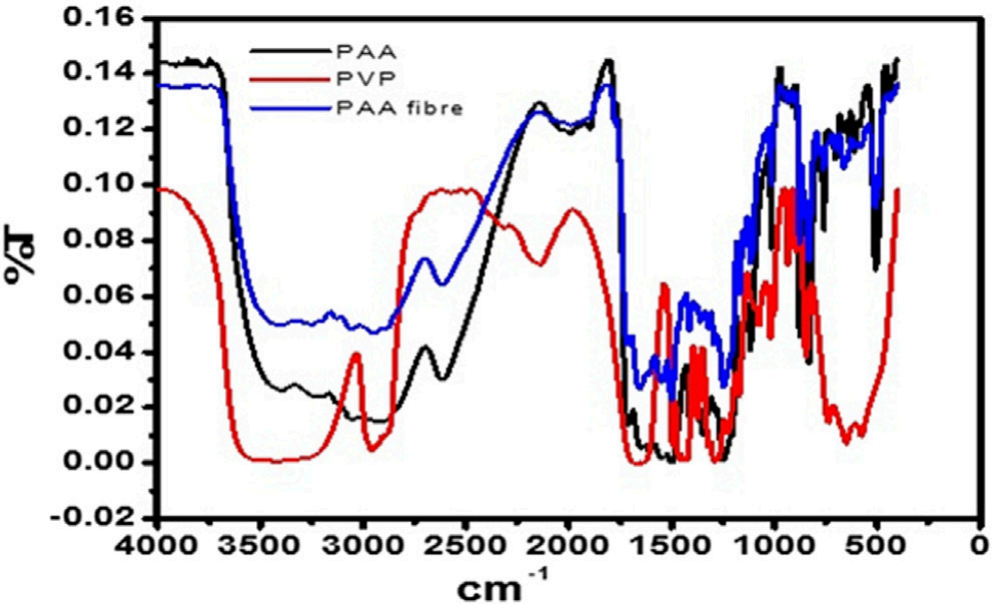
FTIR spectrum of pure PAA powder, PVP, and PAA nanofibers.

The free-standing nanofibers were collected on aluminum foil. FTIR spectra (Figure 2) of PAA powder (chemically synthesized) and stand-alone nanofibers were recorded over the range of 4,000 cm^−1^ to 1,000 cm^−1^, from prepared potassium bromide (KBr) pellets. The FTIR spectra of the PAA nanofibers and chemically synthesized PAA were found to be in good agreement with literature reports (Noah et al., 2012; Hess et al., 2014; Aduba et al., 2015; Lubasova et al., 2015). The absorption bands that occur at around 3,254 cm^−1^, 1,649 cm^−1^, and 1,392 cm^−1^ indicate the presence of the amide group, whereas the bands occurring at around 2,612 cm^−1^ (broad) were assigned to the vibrational modes of carboxylic acid. A spike appearing at 3,049 cm^−1^ was assigned to the NH stretching vibration. The strong peak at around 1,239 cm^−1^ was indicative of the stretching vibrations of the ether group. The FTIR spectra confirmed the integrity of the PAA prepared as nanofibers from electrospinning, by comparison with literature reports as well as with the FTIR data obtained for the chemically synthesized PAA, also done in this work.

### Scanning Microscopy Characterization

**FIGURE 3:**
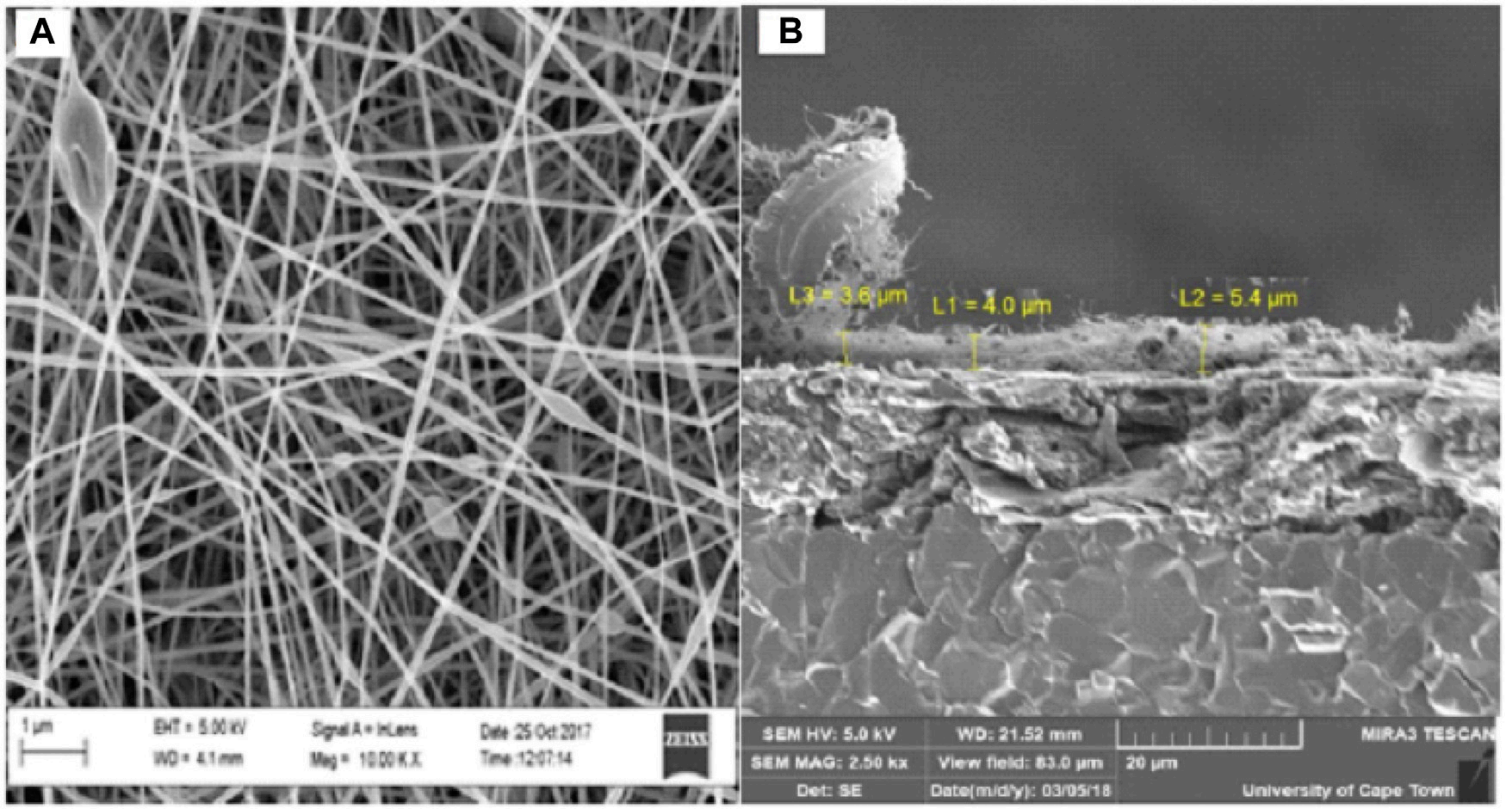
HRSEM **(A)** at 100,00x and cross-sectional SEM **(B)** at 5,000x images of PAA nanofibers.

The high-resolution scanning electron microscope (HRSEM) provides electron backscattering images of the deposited spun nanofibers directly onto a screen-printed carbon electrode (SPCE). SEM images of the PAA nanofibers were identified as an entwined network of nanoscale dimension fibers (Figure 3A). The diameters of the nanofibers shown in these SEM images were estimated at 1–100 nm. The cross-sectional SEM in Figure 3B showed that nanofibers had an average layer thickness of 4.3 µm (*n* = 4). Average layer thickness for electrodeposited PAA films was measured to be 17.63 ųm (*n* = 4, five scans) and 26.54 µm (*n* = 4, 20 scans). The increased layer thickness is due to the space trapped within the nanofiber networks, resulting in a highly porous material with enhanced surface area and robustness. Thus, electrospinning is able to produce intact, mechanically stable, and high–surface area nanofiber networks spun directly onto the working electrode surface of the commercial SPCE. The efficient control over the nanomaterial structure and deposition demonstrated here with the electrospinning of PAA is a major advantage in the design of highly reactive electrocatalysts for a wide range of applications.

### Atomic Force Microscopy

**FIGURE 4:**
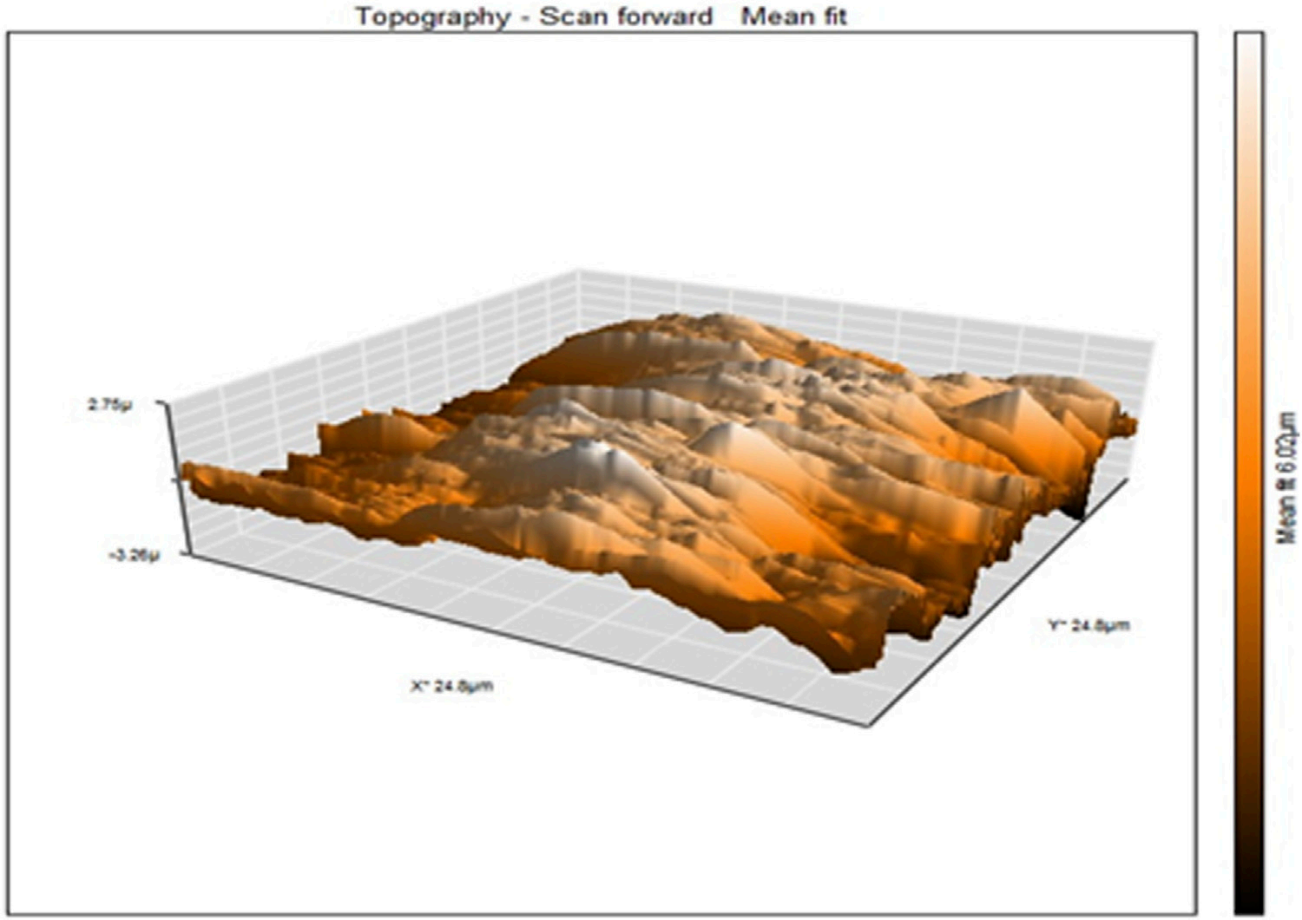
AFM topographical images of electrospun PAA nanofibers.

The atomic force microscopy (AFM) was used to obtain topographical images in the contact mode of PAA nanofibers (Figure 4), which reports the frequency shift (d*f* = *f* − *f*_0_) as a function of the X–Y variation in height without regulation in the *z*-direction (Zamfir et al., 2016). A sample with height and depth shows the distance variation between the tip–apex and the sample for a scan of the sample along the *x*–*y* direction without height regulation in the *z*-direction. The average height distribution of PAA nanofibers was measured as 6.02 µm.

The AFM provides a top-down force sensing measurement of depth. The HRSEM provides surface imaging of the nanofibers in a similar top-down approach but with actual surface feature data generated from backscattered electron imaging. The HRSEM identified the structural arrangement of the nanofibers on the electrode and showed a controlled mesh of nanofibers. The cross-sectional SEM imaging of these nanofibers provided a deep distribution profile of the nanofibers and the entrapped pores between them, showing clearly that the nanofibers have a very dense packing close to the electrode surface, becoming porous as we move away from the electrode surface. This supports the characterization of the deposited nanofibers as a 3D network at the electrode surface from which diffusional properties were assessed.

### Nitrogen Adsorption–Desorption

Isothermal N_2_ measurements were used to study the surface structures of the freestanding polyamic acid (PAA) nanofibers. Based on the BET measurements [see Supplementary Figure SI.3)], the surface area of the PAA nanofibers was found to be 13.9 m^2^/g, comparable to the surface area of synthesized carbon-based nanomaterials such as multi-walled carbon nanotubes with BET surface area ranging from nine up to 500 m^2^/g (Lehman et al., 2011; Tetana et al., 2012).

Cyclic voltammetry of the PAA/SPCE prepared by electrospinning PAA nanofibers directly onto a screen-printed electrode (SPCE) was recorded at scan rates ranging from 10 to 100 mV/s. The redox behavior of PAA nanofibers observed from CV (see Supplementary Figure SI.4) was characteristic of PAA electrochemistry reported for PAA thin films and chemically synthesized PAA powders (Andreescu et al., 2005; Hamnca et al., 2017). PAA nanofiber electrodes showed two anodic peaks at 73 and 350 mV, respectively, with two cathodic peaks at 11 and 268 mV, vs Ag/AgCl. The peak currents reported for these peaks as a function of increasing scan rate between 10 and 100 mV/s showed a linear dependency, with a peak separation of 62 mV supporting a conclusion of a diffusion-controlled reversible process. The porous nature of the nanofiber arrangement, evidenced by cross-sectional SEM, allows for diffusion of ions into these spaces as well as electron diffusion within the polymer nanofiber network. This supports the characterization of the deposited nanofibers as a 3D network at the electrode surface from which diffusional properties were assessed.

The PAA nanofibers displayed enhanced peak resolution in terms of peak shape and redox current intensity, attributed directly due to the high surface area and porosity of the spun fiber networks. The number of electrons transferred was calculated from peak a and a^,^ in the CV data collected at PAA/SPCE using the following equation:

$$\mathrm{Ep-E}p_{\mathrm{1/2}}=2.20\;\mathrm{RT/nF=56}\mathrm{.5/n,}$$ (1)

where Ep is the maximum peak potential, Ep_1/2_ is half maximum peak potential, R is the gas constant (8.314 j.mol.k^−1^), T represents the absolute temperature (298 of the gas system), n is the number of electrons, and F is the Faraday constant (96,584 C/mol). The a/a redox chemistry was found to be a one-electron transfer process associated with the amine functionality of surface bound PAA/SPCE. The surface concentrations of physically adsorbed PAA nanofibers onto the SPCE was estimated from the plot of peak current vs potential at a scan of 50 mV/s (see Supplementary Figure SI.5) using the following equation (Brown–Anson model) (Brown and Anson, 1977):

$$\mathrm{Ip}=n^{2}F^{2}\mathrm{I*Av/4RT,}$$ (2)

where Ip is the cathodic/anodic peak current at a different scan rate, A was taken as the surface area of the unmodified electrode (0.13 cm^2^), v is the scan rate (V/s), I^*^ is the surface concentration (mol cm^−2^), and F, R, and T are the same as in Eq. 1. The surface concentration was calculated to be 2.14 × 10^−6^ mol/cm^2^. The Randles–Sevcik plot (see Supplementary Figure SI.4) was used to calculate the diffusion coefficient (D_e_) of the screen-printed carbon–PAA nanofiber electrode. The diffusion coefficient (D_e)_ of the SPCE–PAA nanofiber electrode was calculated to be 9.43 × 10^−7^ cm^−2^/s. The values reported the literature for diffusion-controlled behavior of PAA thin film–coated electrode surfaces are 6.35 × 10^−7^ cm^2^/s (electrodeposited using cyclic voltammetry onto a gold electrode between −500 and 1,000 mV using 50 cycles at a scan rate of 50 mV/s), 7.10 × 10^−6^ cm^2^/s (electrodeposited onto a glassy carbon electrode using 20 cycles between −400 mV and +600 mV at a scan rate of 50 mV/s), and 5.25 × 10^−6^cm^2^ (electrochemically deposited onto a glass carbon electrode using five cycles between −1,000 mV and +1,000 mV at a scan rate of 50 mV/s (Noah et al., 2012; Hess et al., 2014; Ngema et al., 2019). The diffusion coefficient calculated in this work falls in the range of the reported values for PAA-coated electrodes.

### Electroanalytical Profiling of Sulfonamides

**FIGURE 5:**
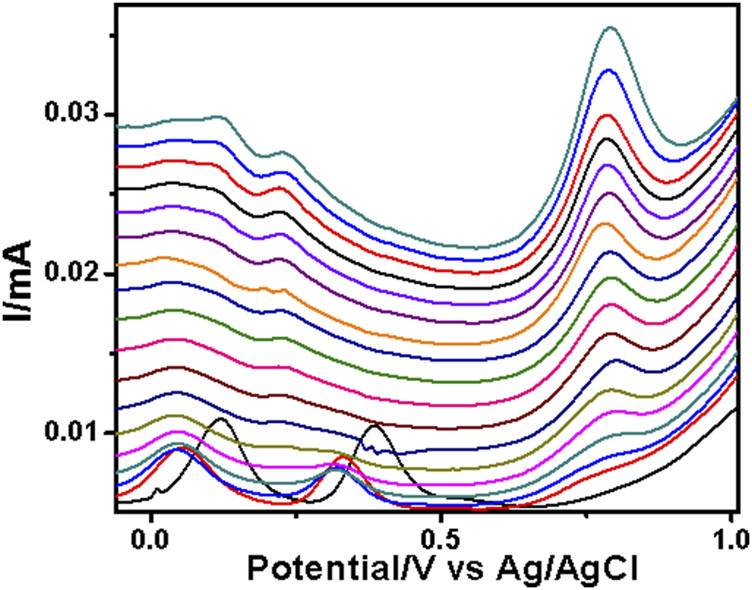
SWV analysis of the sulfadiazine at the PAA nanofiber–modified screen-printed electrode (SPCE) in 0.1 M Tris–HCl with concentrations ranging from 25 to 250 µM at 50 mVs.

**FIGURE 6:**
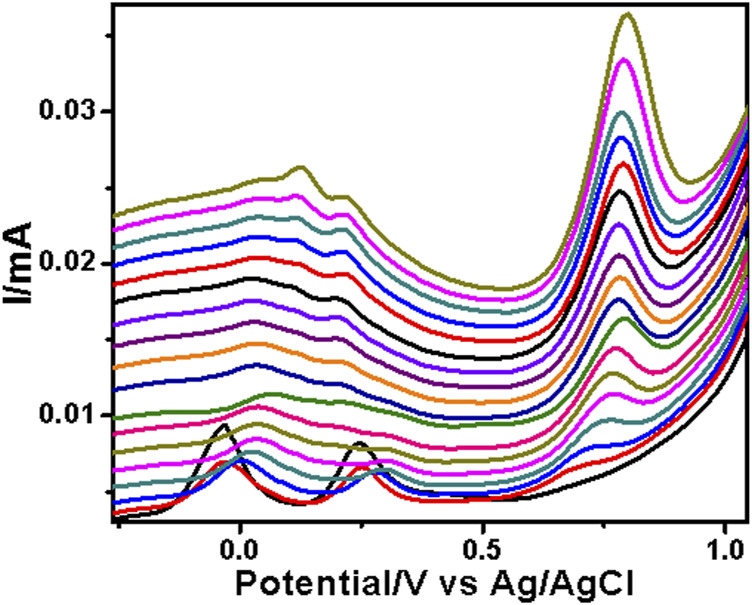
SWV analysis of the sulfamethazine at the PAA nanofiber–modified screen-printed electrode (SPCE) in 0.1 M Tris–HCl with concentrations ranging from 25 to 250 µM at 50 mVs.

The PAA nanofiber modified screen-printed electrodes were used for the analytical reporting of sulfonamides in aqueous medium. A well-defined peak was observed at 0.79 V vs. Ag/AgCl (Figure 5) for sulfadiazine oxidation and at 0.78 V vs. Ag/AgCl (Figure 6) for sulfamethazine oxidation. The sulfonamide functionality typically reports an oxidation peak assigned to the oxidation of the para position –NH_2_ group (Voorhies and Adams, 1958), at potentials ranging from 0.70 to 1.02 V vs. Ag/AgCl (Fotouhi and Zabeti, 2014; Ebrahimi et al., 2017; Su and Cheng, 2018). The sulfonamides evaluated in this work showed oxidation peaks between 0.75 and 0.78 V vs. Ag/AgCl, depending on the specific sulfonamide drug investigated. The oxidation current is due to the para -NH_2_ group, common to all of the sulfonamide drugs evaluated. The small shifts in potential are due to the polymer nanofibers, which attenuates the electron transfer.

**FIGURE 7:**
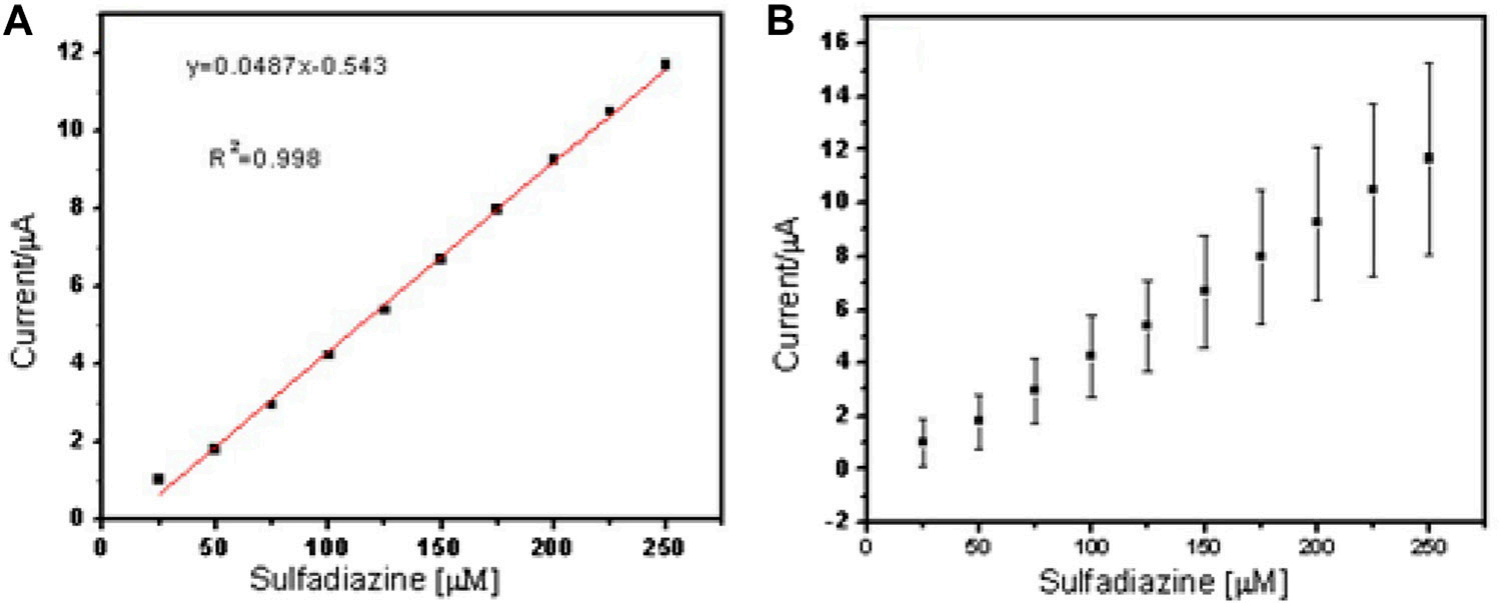
Linear regression plot of current against concentration of sulfadiazine **(A)** and **(B)** standard error bar plot from the standard deviation (*n* = 3).

**FIGURE 8:**
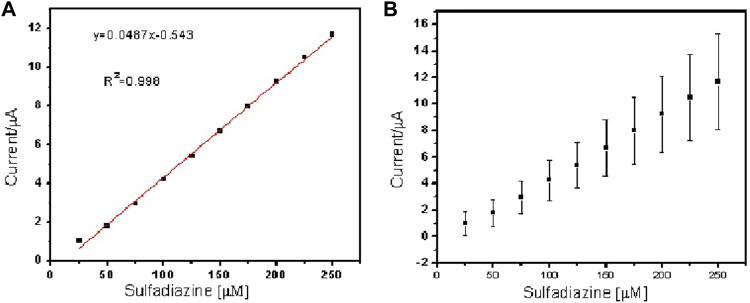
Linear regression plot of current against concentration of sulfamethazine **(A)** and **(B)** standard error bar plot from the standard deviation (*n* = 3).

The two peaks observed at −0.03 V vs. Ag/AgCl and 0.25 V vs. Ag/AgCl were attributed to the porous electrospun polymer material. The sulfadiazine (Figure 7) and sulfamethazine (Figure 8) showed excellent linear regression response for the concentration range evaluated, at the PAA nanofiber–modified electrodes, with *R* ^2^ values of 0.998 and 0.997 (*n* = 3), respectively. The limit of detection (LOD) of sulfadiazine and sulfamethazine was based on the oxidation of the para -NH_2_ group observed at 0.78 V vs Ag/AgCl. The LOD was found to be 8.26 ± 2.07 ųM and 8.81 ± 3.06 μM, respectively. The PAA nanofiber–modified screen-printed electrodes showed good sensitivity toward sulfadiazine (0.05 ± 0.01 μA Lmol^−1^) and sulfamethazine (0.07 ± 0.02 µA μlmol^−1^), which we believe is due to the higher surface area and porosity of the nanofibers network enhancing the catalytic efficiency of the oxidation. However, the reproducibility of the repeated experiments for sulfamethazine was not as good as that for sulfadiazine. Sulfamethazine has methyl group substituents that may affect its alignment at the sensor interface and consequently impact reproducibility.

A comparison of the sensitivity of carbon-based nanomaterial electrodes toward sulfonamide detection showed that carbon nanotubes and PAA electrospun nanofibers outperformed nanoparticles, molecularly imprinted methods, and polymer blends used in the preparation of transducers for the detection of sulfonamides using the SWV method (Sadeghi and Motaharian, 2013; Fotouhi and Zabeti, 2014; Ebrahimi et al., 2017; Su and Cheng, 2018; Urzúa et al., 2018). The implication of the structure and size as opposed to size only, in efficiency of catalysis, is therefore substantiated. The major contribution of this work lies in the successful synthesis and characterization of PAA nanofibers onto SPCE, which to the best of our knowledge has been reported for the first time here. The analytical reporting parameters of LOD and sensitivity are not the lowest reported values but are still competitive compared to other carbon base materials and polymers as the tables demonstrate (Supplementary Tables S1.3, S1.4). In terms of analytical performance enhancement, in this particular application, the surface modification of the SPCE resulted in a wider linear range and lower potential for the analytical peak (Supplementary Tables S1.5.1–3). The sensors therefore present a robust option for sulfonamide detection in aqueous systems.

## Conclusion

The solubility behavior of polymers is crucial in its application as thin film devices such as sensors, photovoltaic cells, and interpenetrating network actuators. We have shown that PAA could be uniformly dispersed in two key organic solvents at relatively high-concentration loading. Subsequently, the critical mass for efficient electrospinning of PAA could be achieved by minimal incorporation of a carrier polymer, which resulted in highly dispersed, uniform nanofibers which could be deposited directly onto the working electrode area of commercial SPCE as well as freestanding nanofibers. The carrier polymer (PVP) did not influence the redox electrochemistry of the PAA as evidenced by the pronounced electrochemical reporting signals obtained. Quantitative analysis of selected sulfonamides corroborated the implication of size and structure on the performance of nano-electrocatalysts. The freestanding PAA nanofibers have potential applications as robust portable electrodes in real-world sensor applications.

## Data Availability

The original contributions presented in the study are included in the article/Supplementary Material; further inquiries can be directed to the corresponding authors.
