# Supplementary material for: Spectroscopy, Morphology, and Electrochemistry of Electrospun Polyamic Acid Nanofibers
Source: Front Chem. 2022 Feb 16;9:782813. doi: 10.3389/fchem.2021.782813 (PMC8889449; doi:10.3389/fchem.2021.782813)
Supplement: Supplementary file 1 [file DataSheet1.pdf]

Appendix A. Supplementary data

Spectroscopy, morphology and electrochemistry of electrospun polyamic acid nanofibers

Siyabulela Hamnca[a,*], Jessica Chamier[b], Sheila Grant[c], Emmanuel Iwuoha[a], and Priscilla Baker[a,*]

[a]SensorLab, Chemistry Department, University of the Western Cape, Bellville, South Africa

[b]HySA Catalysis, Department of Chemical Engineering, University of Cape Town, Rondebosch, Cape Town, South Africa

[c]Biological engineering department , University of Missouri , Columbia , United states of America

Corresponding author : Tel +27 (021) 959 1070

Email addresses : pbaker@uwc.ac.za (PGL Baker), 3055982@myuwc.ac.za (S.Hamnca)

Table S1.1. Solubility evaluation of PAA in different solvents, concentration of PAA and the feasibility of electrospinning the polyamic acid.

**Solvents**	**Concentration of PAA (by wt)**	**Solubility**	**Electrospinning**
Phosphate buffer	1%<br>5%	Not soluble	N/A
Methanol	5%<br>10%	Partially soluble<br>Not soluble	N/A
DMF	5%<br>10%<br>12%<br>20%	Soluble<br>Soluble<br>Soluble<br>Soluble	N/A<br>N/A<br>Spitting<br>Spitting
DMac	5%<br>10%<br>12%<br>20%	Soluble<br>Soluble<br>Soluble<br>Partially soluble/ reached saturation point	N/A<br>N/A<br>Spitting<br>N/A
Acetic acid	5%	Not soluble	N/A
Chloroform	5%	Not soluble	N/A
Water	5%	Not soluble	N/A
DMSO	5%	Not suluble	N/A
Acetone	5%<br>10%	Not soluble<br>Not soluble	N/A
Acetonitrile	5%		N/A
Tetrahydrofuran	5%<br>10%	Partially<br>Not soluble	N/A
Tetrahydrofuran/methanol	5%<br>10%	Partially<br>Not soluble	N/A
NMP	12 %	Soluble	No spitting

Table S1.2. Electrospinning of polyamic acid (PAA) using different parameters of the spinning instrument (IME technologies) and feasibility of using a carrying polymer (PVP).

Solvent	Electrospinning parameters	SEM results

DMF (12% PAA)	Flow rate: 0.2 mL/hr<br>Voltage: 15 kV<br>Distance: 5 cm	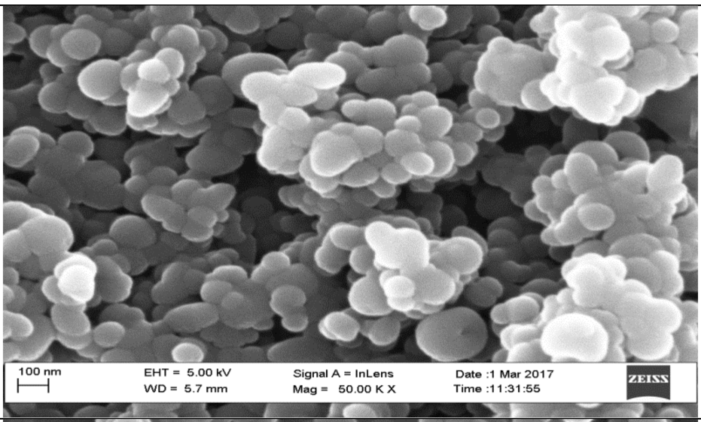
	Flow rate: 0.2 mL/hr<br>Voltage :17 kV<br>Distance: 5 cm	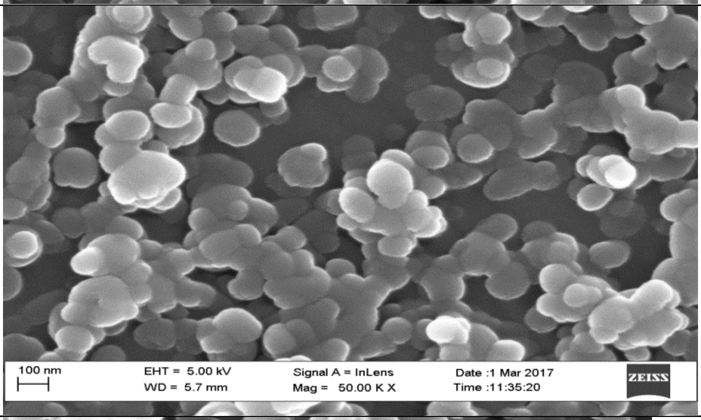
	Flow rate: 0.2 mL/hr<br>Voltage :15 kV<br>Distance: 7.5 cm	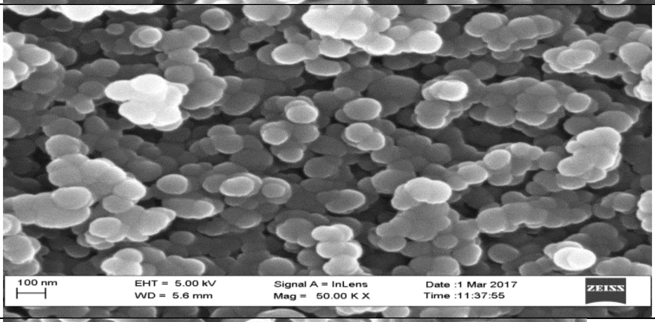
DMac	Flow rate: 0.2 mL/hr<br>Voltage :15 kV<br>Distance: 5 cm	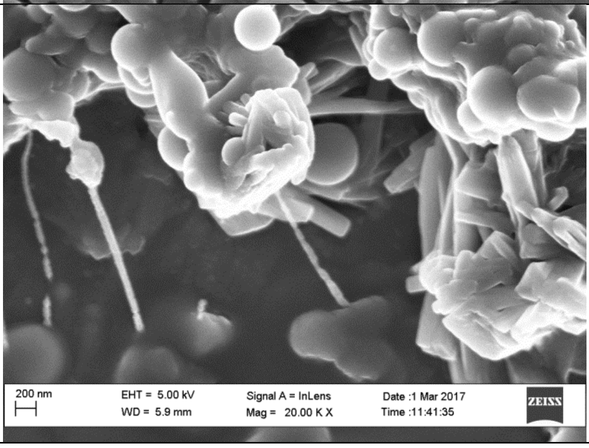

	Flow rate: 0.2 mL/hr<br>Voltage :12 kV<br>Distance: 7.5 cm	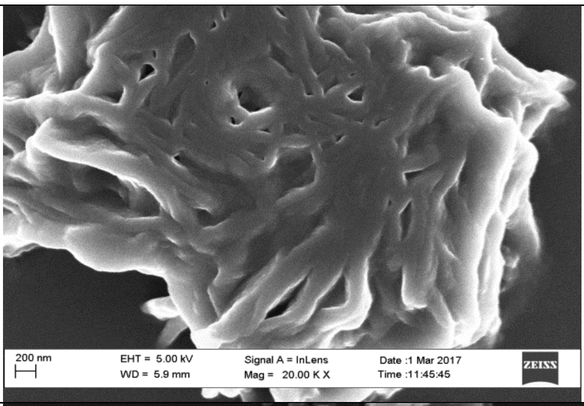
	Flow rate: 0.2 mL/hr<br>Voltage :13 kV<br>Distance: 15 cm	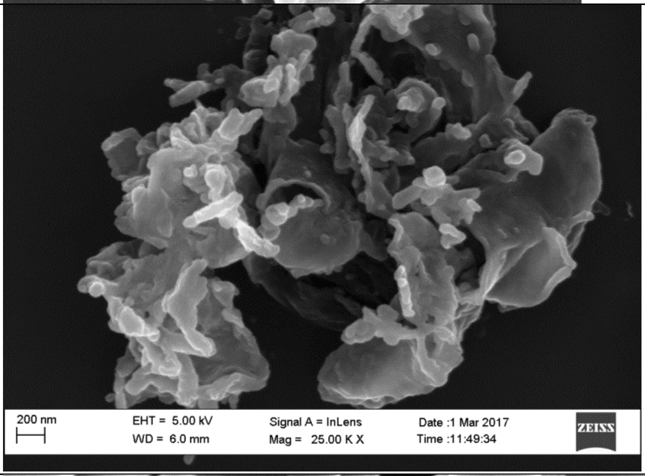
DMF (20% PAA)	Flow rate: 64 µL/hr<br>Voltage :15 kV<br>Distance: 5 cm<br>Humidty control: 21%	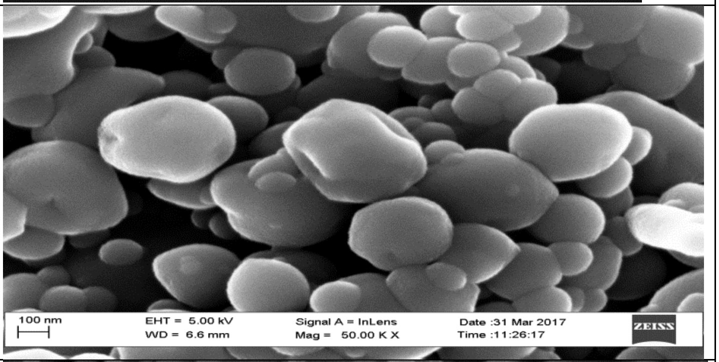
12% PAA+3% PVP (DMF)	Voltage: 16.80 kV<br>Flow rate: 150 µl/hr<br>Distance :15 cm<br>Humidity: 24%<br>Temperature: 23ºC	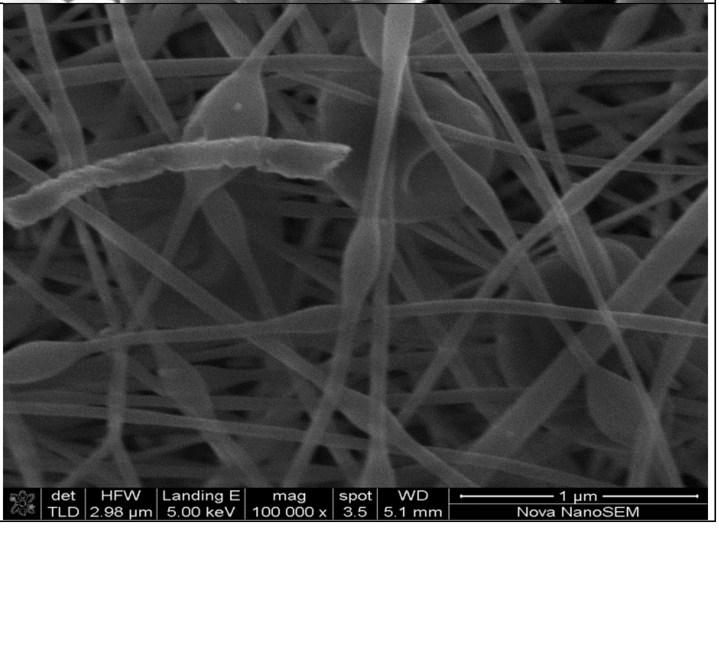

12% PAA+3% PVP (DMac)	Voltage: 11.8 kV<br>Flow rate: 300 µl/hr<br>Distance: 15 cm<br>Humidity: 21%<br>Temperature: 23℃	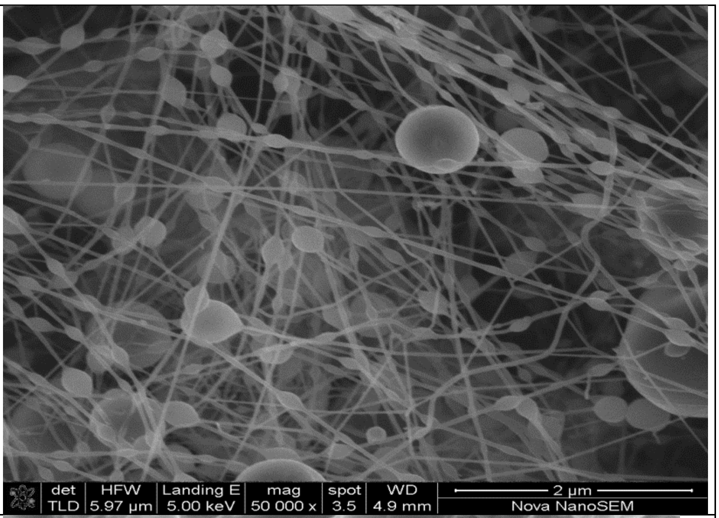
Electrodeposited PAA on Screen printed electrode	0.03 mg/L	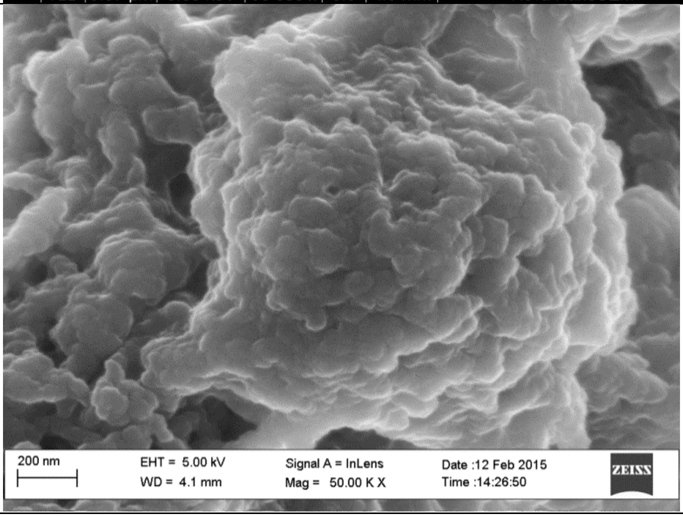

Table S1.3: Comparison of published results with current results from our study

Table S1.3 Electrochemical and analytical data for sulfadiazine detection reported in literature.

**Reference**	**Electrode**	**Method**	**LOD (µM)**	**Peak potentials (V)**	**Sensitivity (µA/ųM)**
Bara et al., 2010	GCE	SWV	10.9	-0.15	0.027

Hong et al., 2011	GCE-MWCNTs	Amperometry	0.21	0.90	0.016
Hong et al., 2012	GCE-MWCNTs –nafion poly(co^{11})	Amperometry	0.71	0.90	0.04
Fatouhi et al., 2013	GCE-MWCNTs	Chrono amperometry	7.10	0.98	0.03
Sadeghi and Motaharian. 2013	CPEs-MIP	DPV	0.14	0.92	4.22
Ebrahim et al., 2017	CPEs-Ceri NPs nanocomposite	FFTSWV	0.17	0.90	0.70
Hong and Ma, 2017	GCE-MWCNT-PSS	Amperometry	0.6	0.95	0.0054
This work	SPCE/PAA nanofibres	SWV	8.81	0.79	0.059
This work	SPCE/PAA nanofibres	Amperometry	3.06	0.79	0.061

Table S1.4 Electrochemical and analytical data for sulfadiazine detection reported in literature.

**Reference**	**Electrode**	**Method**	**LOD (μM)**	**Potential (V)**	**Sensitivity (μA/ųM)**

Özkorucuklu et al. 2008	PGE-OPPy	DPV	0.359	1.15	0.013
Souza et al,.2008	BBD	SWV	1.150	1.10	0.83
Andrade et al.2009	BBD	DPV	0.014	0.92	nr
Joseph and Kumar,.2010	CPE- TMHPP Cu (II)	DPV	0.005	-0.14	nr
Calaca et al. 2014	GCE	SWV	8.560	0.96	0.034
Román et al. 2016	SPCE-Au-TRY	Amperometry	22.60	0.90	0.120
Sgobbi et al. 2016	SPE-MWCNT/PBnc	DPV	0.038	0.58	0.039
Tacco et al. 2018	PDA-MIP	Amperometry	0.800	1.30	nr
Balasubramanian et al.2018	GCE-gC3N4/ZnO nanocomposite	Amperometry	0.007	0.88	0.0031
This work	SPCE/PAA	SWV	8.26	0.79	0.055
This work	SPCE/PAA	Amperometry	1.79	0.79	0,061

nr=not reported

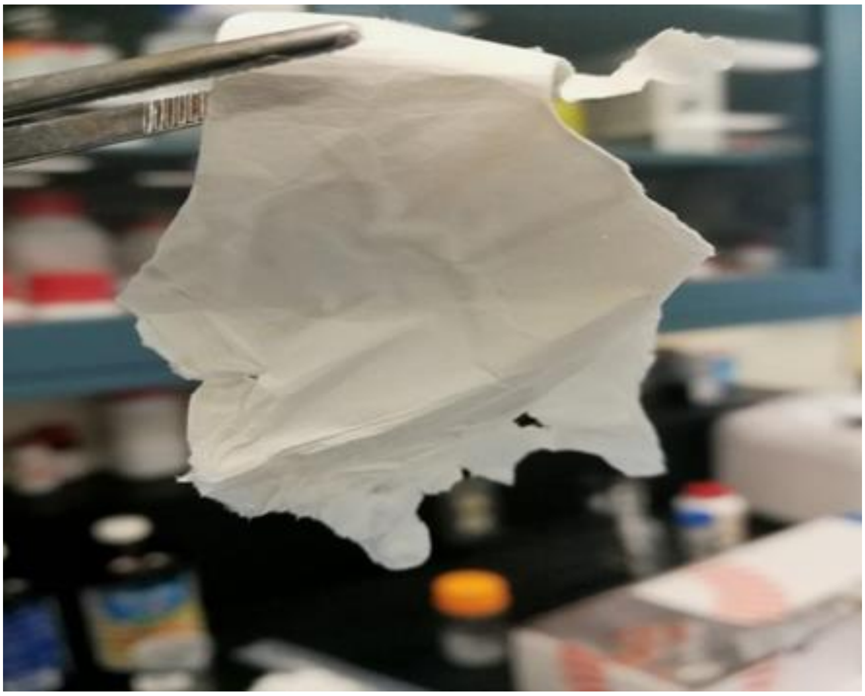

Fig.SI.1. An image of freestanding PAA nanofibers obtained from the custom designed electrospinng instrument in the biological engineering department (University of Missouri, Columbia, MO, USA).

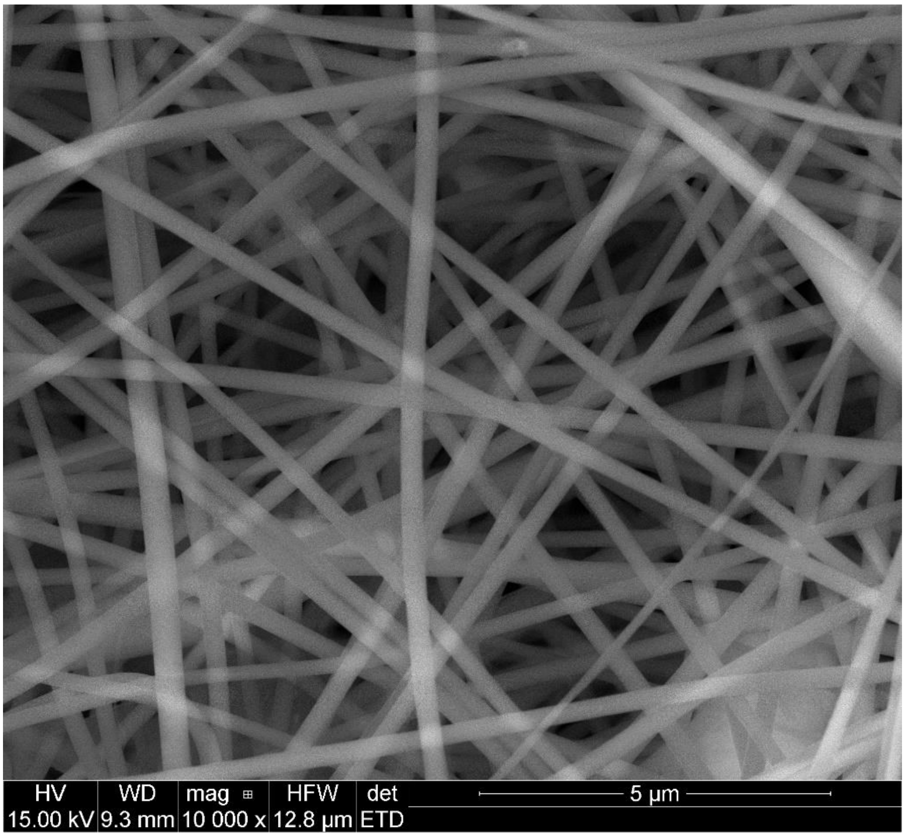

Fig.SI.2. HRSEM image of freestanding PAA nanofibers obtained from the custom designed electrospinng instrument in the biological engineering department (University of Missouri, Columbia, MO, USA).

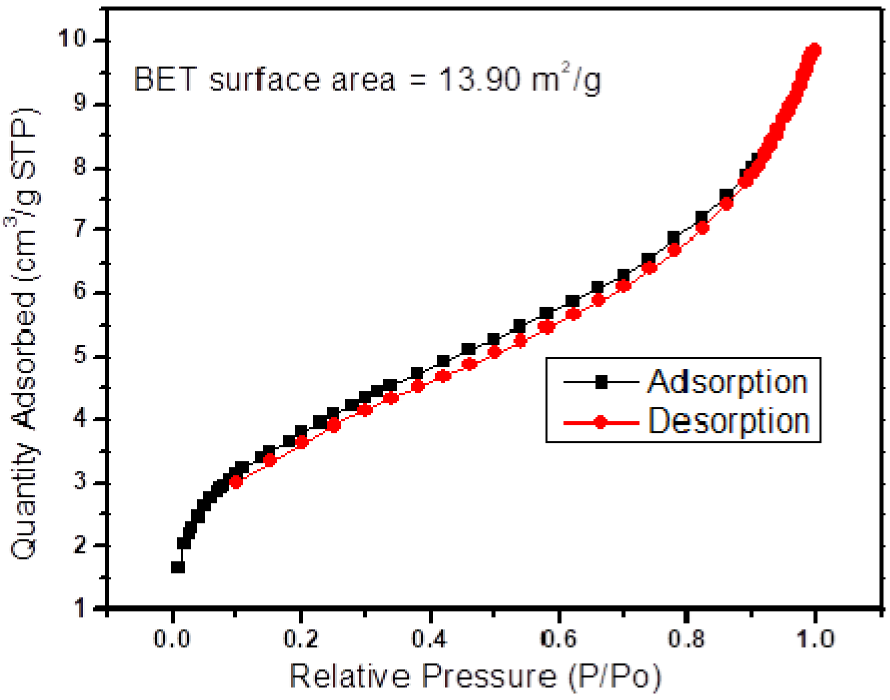

Fig.SI.3. Nitrogen sorption isotherm of PAA nanofibers

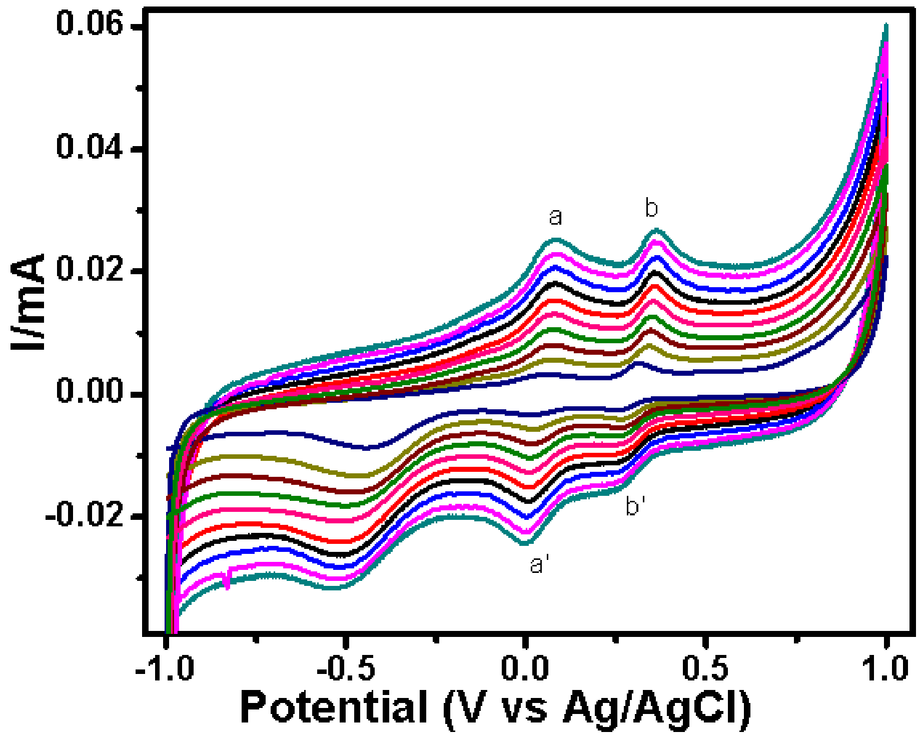

Fig.SI.4. Cyclic voltammogram of screen-printed –PAA nanofiber modified electrode in 0.1 M phosphate buffer (pH 7.0) at different scan rates (10 to 100 mV/s).

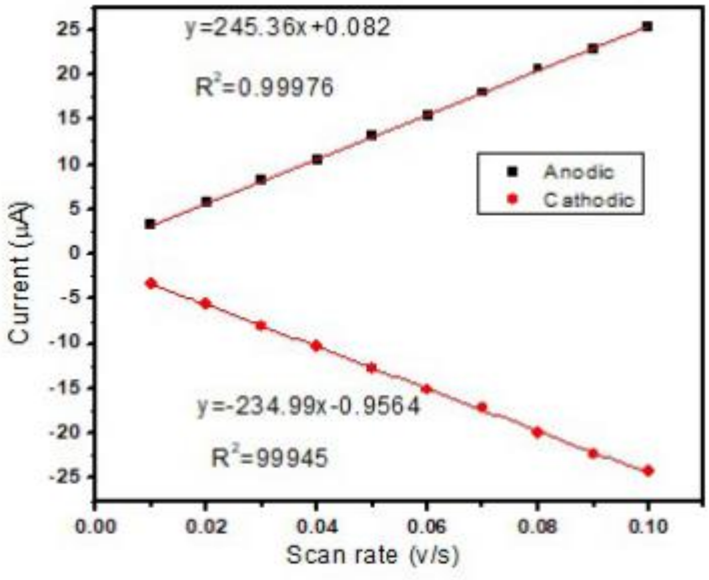

Fig.SI.5. Brown-Anson plot

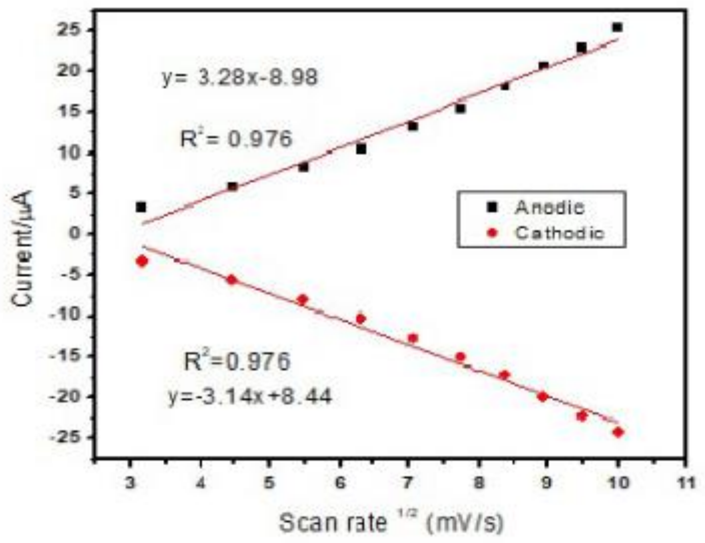

Fig. SI. 6. Randles-Sevick plot.

Table S1.5.1 Peak potential and analytical performance summary of the selected sulphonamides at the unmodified SPCE.

**Analytes**	**Peak potential (V)**	**LOD**	**LOQ**	**Sensitivity (Aµ µM^{-1})**
**Sulfadiazine**	0.80 V	4.07 µM	12.34 µM	0.043
**Sulfamethazine**	0.80 V	1.94 µM	5.98 µM	0.037

Table S1.5.2. Analytical parameters at the SPCE/PAA nanofiber modified electrode as determined by SWV.

Sulfonamides	Peak potentials (V)	Linear range (ųM)	Sensitivity (µA.ųM^{-1})	LOD (ųM)	LOQ (ųM)
Sulfadiazine	0.79	25-250	0.055	8.26	25.04
Sulfamethazine	0.78	25-250	0.059	8.81	26.70

Table S1.5.3 Analytical data for detection of sulphonamides at SPCE/PAA nanofiber sensors using amperommetry

**Sulphonamides**	**Linear range (µM)**	**LOD (µM)**	**LOQ (µM)**	**Sensitivity (µA µM)**
**Sulfadiazine**	25-100	1.79	5.43	0.061
**Sulfamethazine**	12.5-175	3.06	9.27	0.061
